# Structural and Chemical Profiles of *Myrcia splendens* (Myrtaceae) Leaves Under the Influence of the Galling *Nexothrips* sp. (Thysanoptera)

**DOI:** 10.3389/fpls.2018.01521

**Published:** 2018-11-06

**Authors:** Nina Castro Jorge, Érica A. Souza-Silva, Danielle Ramos Alvarenga, Giovanni Saboia, Geraldo Luiz Gonçalves Soares, Cláudia Alcaraz Zini, Adriano Cavalleri, Rosy Mary Santos Isaias

**Affiliations:** ^1^Laboratório de Anatomia Vegetal, Departamento de Botânica, Instituto de Ciências Biológicas, Universidade Federal de Minas Gerais, Belo Horizonte, Brazil; ^2^Departamento de Química, Instituto de Ciências Ambientais, Químicas e Farmacêuticas, Universidade Federal de São Paulo, UNIFESP, Diadema, Brazil; ^3^Laboratório de Química Analítica Ambiental e Oleoquímica, Departamento de Química Inorgânica, Instituto de Química, Universidade Federal do Rio Grande do Sul, Porto Alegre, Brazil; ^4^Laboratório de Ecologia Química e Quimiotaxonomia, Departamento de Botânica, Instituto de Biociências, Universidade Federal do Rio Grande do Sul, Porto Alegre, Brazil; ^5^Instituto de Ciências Biológicas, Universidade Federal do Rio Grande, São Lourenço do Sul, Brazil

**Keywords:** methyl salicylate, *Myrcia*-thrips system, plant–insect interactions, rolling galls, volatiles

## Abstract

Thysanoptera-induced galls commonly culminate in simple folding or rolling leaf gall morphotypes. Most of these galls are induced by members of the suborder Tubulifera, with only a few species of the suborder Terebrantia being reported as gall inducers. The Terebrantia, as most of the gall inducers, manipulates the host plant cellular communication system, and induces anatomical and biochemical changes in its host plant. In an effort to keep its homeostasis, the host plant reacts to the stimuli of the galling insect and triggers chemical signaling processes. In contrast to free-living herbivores, the signaling processes involving galling herbivores and their host plants are practically unknown. Current investigation was performed into two steps: first, we set the structural profile of non-galled and galled leaves, and looked forward to find potential alterations due to gall induction by an undescribed species of *Nexothrips* (suborder Terebrantia) on *Myrcia splendens*. Once oil glands had been altered in size and number, the second step was the investigation of the chemical profile of three tissue samples: (1) non-galled leaves of a control individual, (2) non-galled leaves of galled plants, and (3) galls. This third sample was divided into two groups: (3.1) galls from which the inducing thrips were manually removed and (3.2) galls macerated with the inducing thrips inside. The chemical profile was performed by gas chromatography/ mass spectrometric detector after headspace solid-phase extraction. The galling activity of the *Nexothrips* sp. on M. *splendens* culminates in mesophyll compactness interspersed to diminutive hypersensitive spots, development of air cavities, and the increase in size and number of the secretory glands. Seventy-two compounds were completely identified in the volatile profile of the three samples, from which, sesquiterpenes and aldehydes, pertaining to the “green leaf volatile” (GLVs) class, are the most abundant. The rare event of gall induction by a Terebrantia revealed discrete alterations toward leaf rolling, and indicated quantitative differences related to the plant bioactivity manipulated by the galling thrips. Also, the content of methyl salicylate has varied and has been considered a potential biomarker of plant resistance stimulated as a long-distance effect on *M.* *splendens* individuals.

## Introduction

Among the galling insects, Thysanoptera, commonly known as thrips, are suckers and induce their galls (Meyer, 1987) by means of chemical and/or mechanical stimuli, and alter the development of their host plant tissues (Mani, 1964; Hori, 1992). Galls result from the interaction between a galling organism and its host plant, and demand a high complex and intimate interaction between the associated species (Shorthouse et al., 2005; Raman, 2007). The galling organism manipulates the cellular communication system of the host plant by suppressing its defenses (Oates et al., 2016), and induces anatomical and biochemical changes in the host plant (Raman et al., 2005). Previous studies show that galling insects manipulate plant cells and tissues by the interaction between secondary metabolism and phytohormones (Bedetti et al., 2014). Also, gall induction impairs redox homeostasis, and the accumulation of reactive oxygen species in cell walls is responsible for cell wall loosening and consequent cell redifferentiation and hypertrophy (Isaias et al., 2015). Both of these processes are commonly observed during gall growth and development. Nevertheless, how the insect is able to achieve such an extraordinary level of control over its host plant is perhaps the most intriguing question surrounding plant-galling insect interaction (Oates et al., 2016), and has not been fully described yet.

Along the process of gall induction and the establishment of the galling organism within plant tissues, the plant reacts to the presence of the parasite and chemical signaling mechanisms initiate. Such phenomenon of chemical signaling on plant-herbivore interactions has been widely explored for free-living insects (Rosenthal and Berenbaum, 1992; Dicke et al., 1993), but the signaling mediated by volatile secondary metabolites between galling insects and their host plants is practically unknown (Damasceno et al., 2010).

Gall induction may stimulate the neo-synthesis of secondary metabolites (Oliveira et al., 2006; Guedes et al., 2016) or the standard synthesis of both primary and secondary metabolites may be maintained, but their accumulation is translocated to specific gall tissue compartments (Carneiro et al., 2014; Bragança et al., 2017). Host plants with high potential for the production of volatiles, such as *Myrcia splendens* (Myrtaceae), may come up with novelties regarding the chemical profile of primary metabolites and their involvement in biotic association. The volatile content of the oil glands of *M. splendens* leaves has 95% of sesquiterpenes (Cole et al., 2008; Nakamura et al., 2010), mostly composed of hydrocarbons and oxygenated sesquiterpenes (Cole et al., 2008), this volatile profile can be altered after gall induction.

Our study focuses on a rolling gall morphotype induced by a tiny Thripinae, an undescribed species of *Nexothrips* (suborder Terebrantia) on *M. splendens* (Sw) DC, and it aimed to (1) characterize gall anatomical structure to elucidate how the host plant cell an tissue responses lead to the rolling of leaf lamina; (2) quantify the number and area of the essential oil-producing glands in order to determine whether the gall induction alters the host leaf potential for the production of volatiles; and (3) trace the composition of the volatile compounds emitted by non-galled leaves of a plant totally free of galls (the control individual), by non-galled leaves of galled plants, and by *Nexothrips* sp. galls to detect possible biomarkers of the biotic stress related to gall induction and establishment.

## Materials and Methods

Non-galled leaves and rolling galls were collected from a population of *M. splendens* (Sw.) DC. in Serra Verde State Park (Parque Estadual Serra Verde, PESV), Belo Horizonte, Minas Gerais, Brazil (19°47′21.8″S 43°57′34.4″W). The characterization of the gall morphotype followed Isaias et al. (2013). Individuals of *M. splendens* (*n* = 20) were tagged, and monitored monthly from February 2015 to March 2016. The gall cycle and the diagnosis of the galled and non-galled condition of the individuals, as well as the occurrence and frequency of the galls were analyzed.

### Anatomical Analysis

For anatomical observations, non-galled leaves and mature rolling galls (*n* ≥ 5) were fixed in FAA (formalin, acetic acid, 50% ethanol, 1:1:18) (Johansen, 1940), dehydrated in an *n*-buthanolic series and embedded in Paraplast^®^ (Kraus and Arduin, 1997). The material was sectioned (12 μm) in a rotatory microtome (Leica 2035 BIOCUT^®^), deparaffinized, and stained in astra blue-safranin 9:1 (v/v) (Bukatsch, 1972, modified to 0.5%). The slides were mounted with varnish Acrilex^®^ (Paiva et al., 2006), and the images were obtained with a photomicroscope (Leica ICC50 HP^®^).

### Scanning Electron Microscopy (SEM)

Non-galled leaves and rolling galls fixed in FAA (Johansen, 1940) were dehydrated in an ethanolic series (Johansen, 1940), critical point dried, mounted on stubs, and covered with 15 nm of gold (Balzers SCD 050) (O’Brien and McCully, 1981). The samples were observed in a scanning electron microscope (JEOL JSM - 6360LV) in the Center of Microscopy at the Universidade Federal de Minas Gerais, Belo Horizonte, Minas Gerais state, Brazil^1^.

### Quantitative Analysis of Glands

For the analysis of oil glands, non-galled leaves (NGL) and mature rolling galls (*n* = 20 per sample) were clarified in 50% potassium hydroxide until complete bleaching, washed in water (three times), stained with 1% safranin in 95% ethanol, and dehydrated in ethanolic series (Bersier and Bocquet, 1960). The slides were mounted with varnish Acrilex^®^ (Paiva et al., 2006), and the images were obtained with a photomicroscope (Leica ICC50 HP^®^). The number of glands per area (8.00/mm^2^) was counted, and the relative area of the glands on non-galled leaves and rolling galls was measured using the AxioVision 7.4 software (Carl Zeiss^®^ Microscopy GmbH, Jena, Germany). Statistical analyzes were performed using the SigmaStat^®^ software, and *t*-test was applied considering *p* ≤ 0.05.

### Profile of Volatile Compounds by HS-SPME-GC/MS

Volatile compounds were analyzed in (1) non-galled leaves (*n* = 5) from an individual (*n* = 1), which did not have galls along the year (the control individual), and (2) non-galled leaves (*n* = 5), and (3) leaf rolling galls (*n* = 5) from young individuals (*n* = 14) of *M. splendens* in vegetative phenophase in July 2016. The samples were collected and immediately placed under dry ice, in the field. They were macerated in liquid nitrogen, powdered, and divided into 3 to 5 vials of 20 ml (25 mg/vial), in the laboratory. The vials were immediately stored in ultrafreezer (-80°C) until analysis.

The samples were divided into four composites, as follows: (1) control individual (Ctrl) – composite of all leaves obtained from the non-galled individual; (2) non-galled leaves (NGL) – composite of the leaves of the fourteen individuals; and (3) leaf-rolling galls – composite of the galls of the fourteen individuals, divided into two groups: (3.1) galls from which the inducing thrips were manually removed (cLRG), for the detection of volatiles exclusive to plant tissues, and (3.2) galls macerated with the inducing thrips inside (LRGwT) for detection of volatile compounds exclusive to the inducing thrips.

Volatile profiles were obtained by extracting the compounds from the samples employing HS-SPME with subsequent analyses by gas chromatography coupled to mass spectrometry (Souza Silva et al., 2017). Briefly, 20 mL vial containing 0.025 g of powdered plant material was taken from the ultrafreezer just before extraction. Immediately, 2 μL of internal standard (aqueous solution of 1,4-cineole at 100 ng μL^-1^) was added to the sample with the use of a 10 μL Hamilton syringe pierced through the vial cap septum, without opening the vial cap. Subsequently, each sample vial was pre-incubated on a home-made heating block at 30 ± 2°C, without agitation, for 30 min prior to exposure of the SPME fiber to the vial HS. After 15 min of extraction, the fiber was desorbed into the GC injector at 240 °C for 15 min. Upon desorption of the volatile compounds, the samples were analyzed by gas chromatography coupled to mass spectrometry (GC/MS) with a Shimadzu 2010Q-Plus GC/MS equipment equipped with a DB-5 column (30 m × 0.25 mm × 0.25 μm).

Peaks in the total ion current chromatograms (TICC) were tentatively identified comparing the experimentally acquired mass spectra and the NIST08 mass spectral library, with minimal mass spectra match threshold of 80%. In addition, retention indices (RI) were determined using data obtained from an *n*-alkane solution (C8–C28), and compared to RI reported in the literature. For sesquiterpenes, whenever an appropriate match between mass spectra library hit and retention index was not found, only the family group was designated, according to their mass spectra fragmentation. Relative amounts, as percentages of each component, were achieved by peak area normalization measured without any correction factor. The response obtained for the internal standard 1,4-cineole, measured as area counts, was utilized to monitor possible drifts in instrumental response (Adams, 2001, 2007; Supplementary Material).

The final data matrix containing the relative percentages of each identified peak was submitted to statistical analysis. All analyses were performed assuming 95% level of confidence (α = 0.05). Since the chromatographic response (area) for each compound had already been normalized, no further data normalization or transformation was performed, and scaling of the data was performed using Pareto scaling (mean-centered and divided by the square root of standard deviation of each variable) in order to give all variables equal weight regardless their absolute value. This procedure is especially useful to generate a sound statistical analysis since the levels of the volatile compounds found may be of very different orders of magnitude. After data pre-processing, Principal Component Analysis (PCA), heat map with hierarchical clustering, and Partial Least Squares Discriminant Analysis (PLS-DA) were performed using web-based metabolomic data processing tool MetaboAnalyst^2^. PCA was used to detect intrinsic clusters and outliers within the data set, while PLS-DA maximized class discrimination.

## Results

### Anatomical Analysis

**FIGURE 1:**
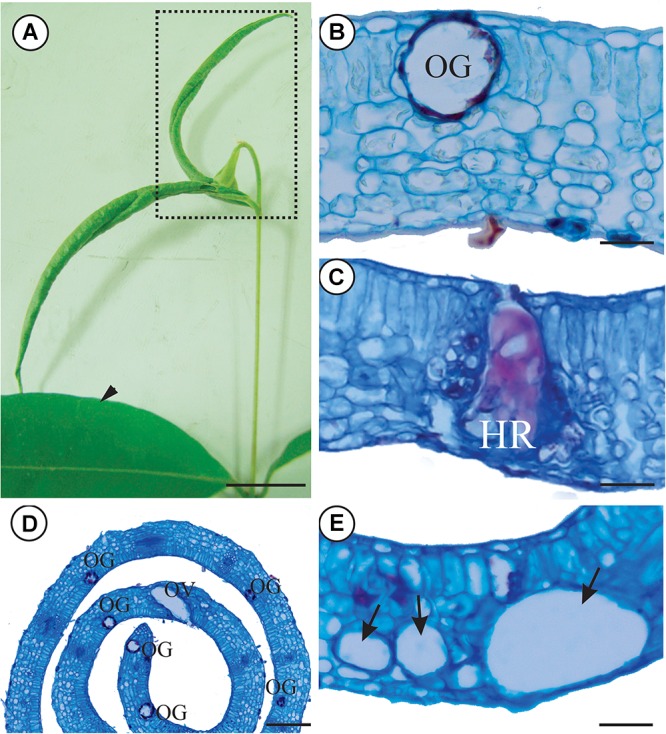
Non-galled leaves and galls induced on leaves of *Myrcia splendens* by an undescribed species of *Nexothrips* (suborder Terebrantia) **(A)** Non-galled leaves and stem branch with leaf rolling galls. **(B)** Anatomy of non-galled leaves, evidencing the mesophyll and oil glands. **(C)** Gall mesophyll, evidencing the hypersensitive reaction in response to the inducer’s feeding activity. **(D)** Anatomy of a gall, evidencing the increased number of secretory cavities. **(E)** Gall mesophyll, evidencing the reduction of intercellular spaces and air cavities. Arrowhead: Non-galled leaves, Dashed: Rolling gall, OG: Oil gland, OV: Oviposition, HR: Hypersensitive area, Arrow: Air cavities Scale bars: **A**: 2 cm, **B**,**C**,**E**: 50 μm; **D**: 200 μm.

The leaves of *M. splendens* in non-galled condition are green (Figure 1A), dorsiventral and hypostomatic. The epidermis is uniseriate, the mesophyll has a 1- layered palisade parenchyma and 7–10 layered spongy parenchyma. Secretory cavities occur all over the mesophyll, and in the midrib cortex (Figure 1B).

**FIGURE 2:**
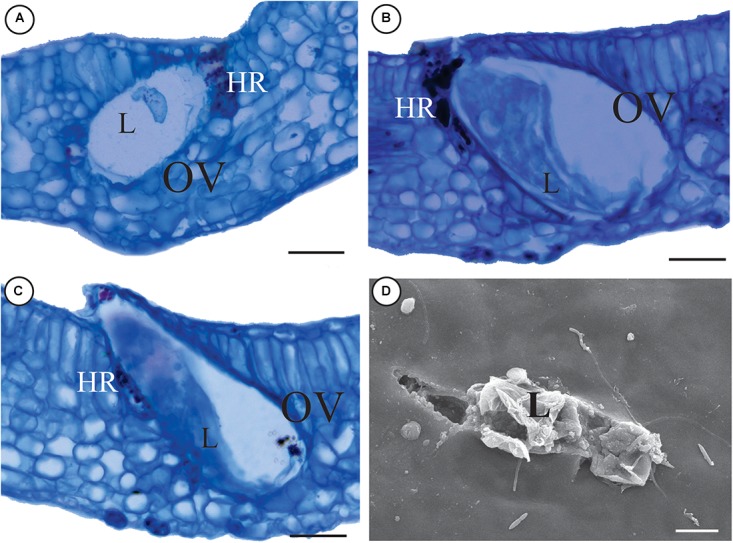
Oviposition inside the tissues of a *M. splendens* rolling gall induced by an undescribed species of *Nexothrips* (suborder Terebrantia). **(A)** Initial development of the larva inside gall tissues. **(B)** Development of the larva. **(C,D)** Larvae hatching out of gall tissues. **(C)** Anatomy of a gall, evidencing hatching out of the leaf tissue. **(D)** MEV slides evidencing the hatching. L: Larva, OV: oviposition, HR: Hypersensitive area. Scale bars: **A–C**: 50 μm; **D**: 10 μm.

The galls are green with the rolling movement of leaf lamina upward, along both sides of the midrib (Figure 1A). Galling thrips in several stages of life occur inside the galls, but parasitoids and predators are rare. Hypersensitive reactive spots form in response to the feeding activity of the thrips in the epidermis and mesophyll (Figure 1C). The number of secretory cavities increases and the cells of the epithelium are hypertrophied (Figure 1D). The spongy parenchyma is 5–7 layered and compact due to a reduction of intercellular spaces. Large air cavities are evident within mesophyll cells (Figure 1E). The oviposition takes place inside leaf tissues, where the larvae develop. Later on, the immature thrips hatch out of the tissues (Figures 2A–D) and start feeding.

### Quantitative Analysis of Glands

**FIGURE 3:**
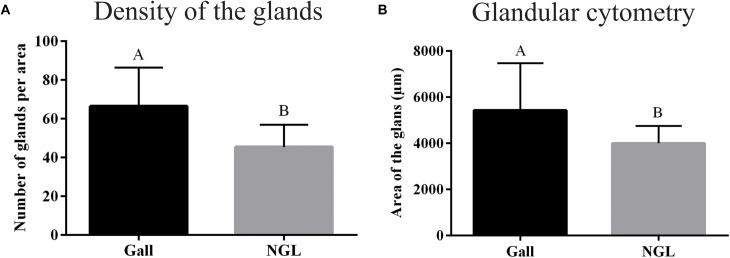
Quantitative analysis of glands. **(A)** Density of the glands per mm^2^. **(B)** Glands area. NGL: Non-galled leaves.

Both the number and the area of the oil glands are statistically different between non-galled leaves and galls. The density of the glands per mm^2^ was higher in galls than in non-galled leaves (*p* < 0.0001) (Figure 3A), and the area of the glands was larger and more variable (*p* = 0.0227) in galls than in non-galled leaves (Figure 3B).

For the quantitative analysis of the oil glands, the ANOVA tests were applied for parametric data, and Kruskal test for non-parametric data.

### Volatile Profile Characterization by HS-SPME-GC/MS

A total of 84 compounds were aligned across all samples. Twelve compounds were tentatively identified only as sesquiterpenes due to their characteristic mass spectra fragmentation pattern, inconsistencies between NIST library hit, experimental retention indexes and literature retention indexes. The complete identification of 72 metabolites was possible according to their retention indexes (Table 1). The 36 sesquiterpenes constitute the major class of the identified compounds, followed by monoterpenes (12), alcohols (10), aldehydes (10), esters (5), aromatic compounds (5), ketones (3), and hydrocarbon (1). Green leaf volatiles (GLVs), comprising low molecular weight oxygenated compounds, amounted to 17 compounds.

**Table 1:** Metabolites tentatively and positively identified in the headspace of galled and non-galled leaves of *Myrcia splendens.*

#	Analyte	Class	RT (min)	RI _exp_	RI _Lit_
1	Ethanol	Alcohol	1.864	636	448
2	1-Penten-3-ol	Alcohol (GLV)	3.894	690	684
3	1-Penten-3-one	Ketone (GLV)	3.945	691	686
4	Pentanal	Aldehyde (GLV)	4.203	698	699
5	3-Buten-1-ol, 3-methyl-	Alcohol (GLV)	5.201	724	724
6	1-Butanol, 3-methyl-	Alcohol (GLV)	5.432	730	733
7	3-Penten-2-one	Ketone (GLV)	5.525	733	733
8	2-Pentenal, (E)-	Aldehyde (GLV)	5.666	737	740
9	2-Pentenal, (Z)-	Aldehyde (GLV)	6.017	746	740
10	1-Pentanol	Alcohol (GLV)	6.715	763	763
11	2-Penten-1-ol, (Z)-	Alcohol (GLV)	6.819	767	767
12	3-Hexenal, (Z)-	Aldehyde (GLV)	7.958	797	796
13	Hexanal	Aldehyde (GLV)	8.02	799	800
14	2-Hexenal, (E)-	Aldehyde (GLV)	10.968	855	854
15	3-Hexen-1-ol, (Z)-	Alcohol (GLV)	11.114	858	857
16	2-Hexen-1-ol, (E)-	Alcohol (GLV)	11.597	866	863
17	1-Hexanol	Alcohol (GLV)	11.718	869	871
18	Heptanal	Aldehyde (GLV)	13.317	899	901
19	α-Thujene	Monoterpene	14.786	925	924
20	α-Pinene	Monoterpene	15.106	931	934
21	Benzaldehyde	Aromatic	16.362	952	960
22	4-Hexen-3-one, 5-methyl-	Ketone	16.499	955	961
23	β-Pinene	Monoterpene	17.491	972	974
24	1-Octen-3-one	Ketone	17.657	975	975
25	1-Octen-3-ol	Alcohol	17.827	978	980
26	5-Hepten-2-one, 6-methyl-	Ketone	18.177	984	985
27	2,4-Heptadienal, (E,E)-	Aldehyde	18.613	992	1011
28	D-Limonene	Monoterpene	20.48	1026	1027
29	Benzyl Alcohol	Aromatic	20.696	1030	1032
30	Benzeneacetaldehyde	Aromatic	21.104	1037	1040
31	β-Ocimene	Monoterpene	21.676	1047	1050
32	Linalool oxide	Monoterpene	22.911	1070	1069
33	α-Terpinolene	Monoterpene	23.783	1086	1088
34	Linalool	Monoterpene	24.431	1097	1097
35	Nonanal	Aldehyde	24.648	1101	1101
36	Phenylethyl Alcohol	Aromatic	24.65	1108	1106
37	*cis*-Pinocarveol	Monoterpene	26.384	1135	1136
38	2,4,6-Octatriene, 2,6-dimethyl-, (E,Z)-	Monoterpene	26.663	1128	1131
39	α-Terpinen-4-ol	Monoterpene	28.432	1174	1178
40	Naphthalene	Aromatic	28.551	1176	1169
41	Butanoic acid, 3-hexenyl ester, (Z)-	Ester	28.999	1185	1184
42	Methyl salicylate	Ester	29.234	1190	1193
43	Octanoic acid, ethyl ester	Ester	29.585	1193	1195
44	Decanal	Aldehyde	29.928	1203	1205
45	Dodecane	Hydrocarbon	29.928	1200	1200
46	β-Cyclocitral	Monoterpene	30.594	1217	1217
47	Citronellic acid, methyl ester	Ester	32.691	1260	1261
48	2,6-Octadienoic acid, 3,7-dimethyl-, methyl ester	Ester	35.709	1323	1323
49	**Sesquiterpene 1**	Sesquiterpene	35.975	1329	n/a
50	**Sesquiterpene 2**	Sesquiterpene	36.113	1331	n/a
51	δ-Elemene	Sesquiterpene	36.504	1340	1339
52	α-Cubebene	Sesquiterpene	37.048	1352	1352
53	Cyclosativene	Sesquiterpene	37.761	1367	1368
54	α-Ylangene	Sesquiterpene	38.041	1373	1373
55	α-Copaene	Sesquiterpene	38.353	1380	1380
56	β-Bourbonene	Sesquiterpene	38.689	1387	1388
57	**Sesquiterpene 3**	Sesquiterpene	38.903	1392	n/a
58	β-Elemene	Sesquiterpene	39.035	1395	1394
59	**Sesquiterpene 4**	Sesquiterpene	39.179	1398	n/a
60	α-Gurjenene	Sesquiterpene	39.796	1412	1413
61	β-Caryophyllene	Sesquiterpene	40.339	1424	1424
62	γ-Elemene	Sesquiterpene	40.721	1433	1433
63	α-Caryophyllene	Sesquiterpene	41.076	1441	1443
64	**Sesquiterpene 5**	Sesquiterpene	41.225	1445	n/a
65	**Sesquiterpene 6**	Sesquiterpene	41.884	1460	n/a
66	γ-Muurolene	Sesquiterpene	42.189	1467	1465
67	δ-Muurolene	Sesquiterpene	42.274	1468	1468
68	α-Muurolene	Sesquiterpene	43.432	1499	1498
69	Germacrene D	Sesquiterpene	43.506	1496	1492
70	γ-Cadinene	Sesquiterpene	43.929	1508	1509
71	σ-Cadinene	Sesquiterpene	44.455	1523	1523
72	δ-Cadinene	Sesquiterpene	44.819	1533	1533
73	**Sesquiterpene 7**	Sesquiterpene	45.069	1540	n/a
74	**Sesquiterpene 8**	Sesquiterpene	45.21	1544	n/a
75	3,7(11)-Selinadiene	Sesquiterpene	45.285	1546	1545
76	Germacrene B	Sesquiterpene	45.441	1551	1553
77	**Sesquiterpene 9**	Sesquiterpene	46.098	1569	n/a
78	Caryophyllene oxide	Sesquiterpene	47.063	1597	1599
79	**Sesquiterpene 10**	Sesquiterpene	47.44	1609	n/a
80	**Sesquiterpene 11**	Sesquiterpene	47.668	1617	n/a
81	τ-Cadinol	Sesquiterpene	48.564	1647	1643
82	α-Cadinol	Sesquiterpene	48.949	1660	1657
83	**Sesquiterpene 12**	Sesquiterpene	50.169	1702	n/a
84	2,6,10-Dodecatrien-1-ol, 3,7,11-trimethyl-, (E,E)	Sesquiterpene	50.712	1723	1722


**FIGURE 4:**
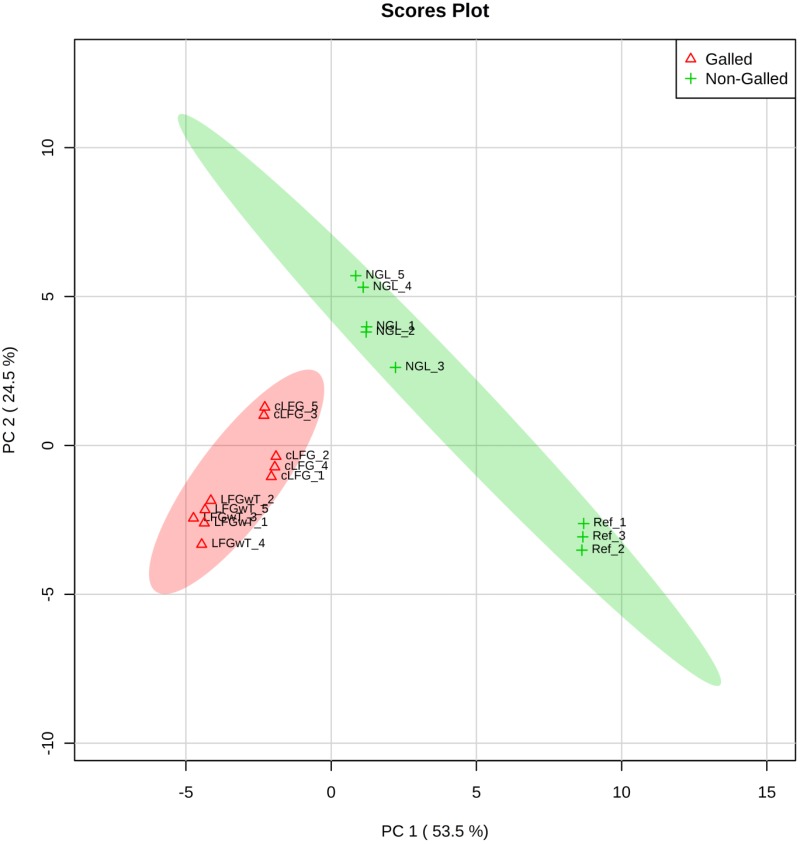
Profile of the distribution of VOCs in non-galled leaves and galls. Plot depicts the distribution of VOC profiles of leaves (non-galled samples, including NGL and Ctrl) and galls (including LRGwT and cLRG samples) over the PCA score plot defined by the first two principal components.

The profiles of volatile organic compounds (VOC) obtained from the four composites could be separated into two classes by the PCA analysis: (1) leaves of the non-galled individual, comprising the control group (Ctrl) together with the non-galled leaves (NGL), and (2) galls with thrips (LRGwT) and galls without thrips (cLRG) (Figure 4).

**FIGURE 5:**
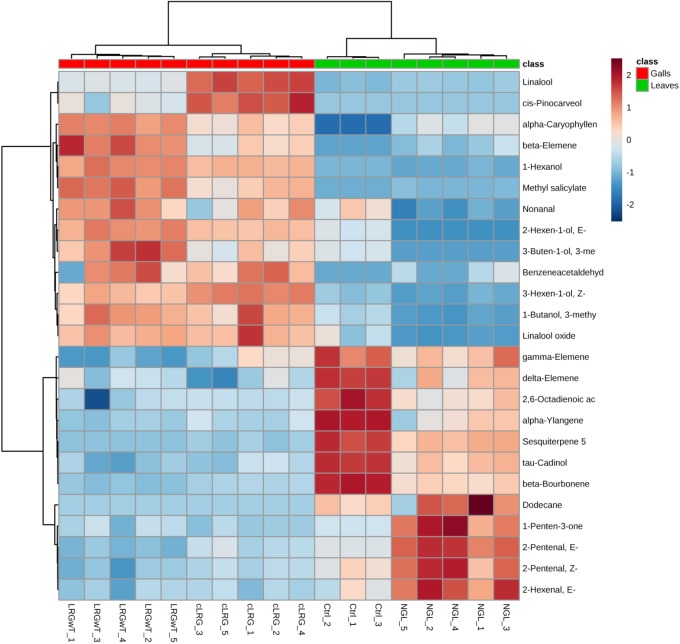
Profile of the distribution of VOCs in non-galled leaves and galls. Plot shows the heat map distribution of the 25 most discriminating VOCs identified by SPME-GC-MS analysis. Color key indicates metabolite expression value, blue: Lowest, red: highest.

**FIGURE 6:**
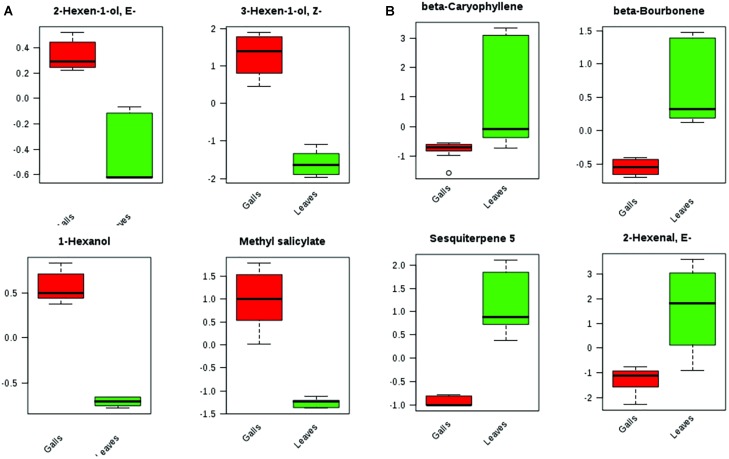
Box plots of top metabolites that significantly differed between leaves and galls. **(A)** VOCs up regulated in gall samples. **(B)** VOCs down regulated in gall samples.

**FIGURE 7:**
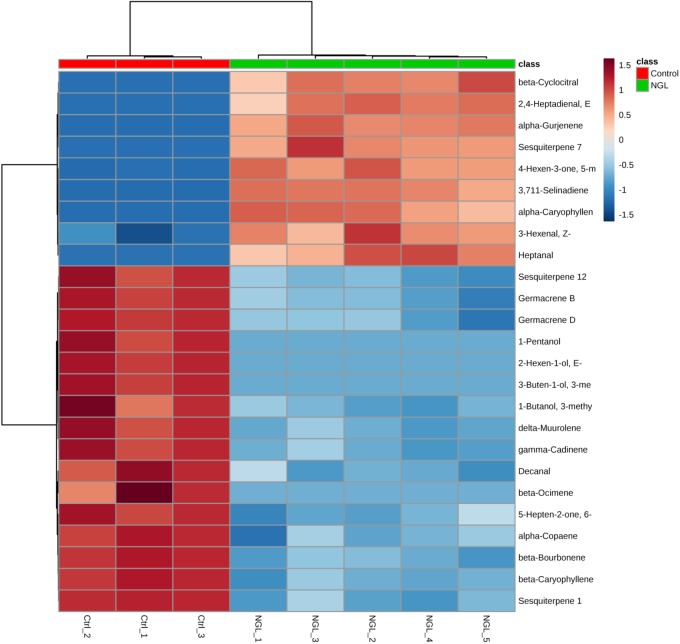
Heat map of non-galled leaves. The heat maps were constructed based on the 25 most discriminating compounds. Compounds identified by HS-SPME-GC/MS. Color key indicates metabolite expression value, blue: Lowest, red: highest.

Even though the four groups could be successfully separated into two classes (PCs 1 and 2 explain 78% of variance in the data), the 25 most discriminating VOCs show that there is a clear distinction between the profile of the groups within a class, i.e., between the Ctrl and the NGL samples, and between the cLRG and the LRGwT (Figure 5). The main compounds responsible for the separation of the groups are 2-*E*-hexenal (#14), sesquiterpene 5 (#64), β-caryophyllene (#61), and β-bourborene (#56) that are upregulated in the leaves of non-galled samples, and methyl salicylate (#42), 3-*Z*-hexen-1-ol (#15), 2-*E*-hexen-1-ol (#16) and 1-hexanol (#17), which are upregulated in the samples of galls (Figure 6). In fact, there were significant distinguishing features between the control sample (Ctrl) and the non-galled leaves of galled individuals (Figure 7). Contrastingly, to the higher levels of sesquiterpenes in the samples of the control individual, there were increased levels of aldehydes in the non-galled samples of galled individuals, mainly of the C6 aldehydes from GLV class.

**FIGURE 8:**
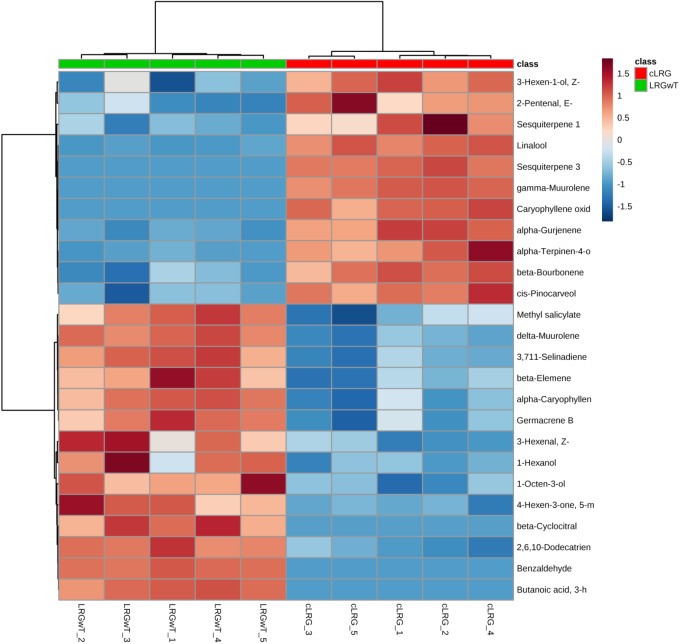
Heat map of galls. The heat maps were constructed based on the 25 most discriminating compounds. Compounds identified by HS-SPME-GC/MS. Color key indicates metabolite expression value, blue: Lowest, red: highest.

In a similar pattern, benzaldehyde (#21) and methyl salicylate (#42) appeared to be upregulated in the LRGwT (galls with inducing thrips inside) composite as compared to the cLRG. Moreover, sesquiterpenes, such as δ-muurolene (#67), β-elemene (#58) and α-caryophyllene (#63), decreased in the composites of galls from which the galling *Nexothrips* were removed (cLRG) (Figure 8).

## Discussion

The first step in current investigation, i.e., the structural profile of non-galled leaves and galls, revealed that the development of the leaf rolling gall morphotype on *M. splendens* results in discrete alterations in epidermis, and in conspicuous alterations in palisade and spongy parenchymas. These alterations lead to a complete rolling of leaf lamina upward.

The mosaic of tissue alterations of *Nexothrips*-induced galls on *M. splendens* with its peculiar air cavities and compactness of spongy parenchyma is not an exclusive feature, for it has been previously described for galls of *Aneurothrips priesneri* Bhatti on *Cordia obliqua* Willd. This pattern seems to be consequence of cell displacement due to the stretching and folding/rolling of leaf lamina throughout gall development (Ananthakrishnan and Raman, 1989). Even though the origin of the stimuli for gall induction remains unknown (Mound and Kranz, 1997), the insect saliva seems to be involved in gall induction and development of thrips-induced galls (Ananthakrishnan and Raman, 1989). Nevertheless, the role of oviposition has not been considered in gall induction of most galling Thysanoptera, since females of suborder Tubulifera lay their eggs externally to plant tissues. Comparatively, the ovipositor of most female Terebrantia is well-developed, and eggs are inserted in a cavity within mesophyll cells. Although this endophytic process has been shown by almost all phytophagous Terebrantia, only three thrips species are reported as capable of causing plant cell responses and gall induction through oviposition (Ananthakrishnan, 1978a,b; Tree and Mound, 2009).

The females of *Nexothrips* oviposit on mature leaves of *M. splendens*, which should be a strategy to avoid the crushing of the egg cavities by the intense cell proliferation common in young developing leaves (Rivnay, 1935). Besides crushing the cavities, the hyperplasia of young leaf tissues should end up pushing eggs out of the leaf, which should not favor the establishment of the galling thrips and gall development. The ability of inducing galls on mature leaves rather than exclusively on young leaves, as is common for most galling insects, guarantees to the individuals of *Nexothrips* sp. a high availability of sites for completing their life cycles. However, the more differentiated is a cell, the less responsive it is. So, inducing galls on mature leaves may impose constraints for the differentiation of high-specialized cells, such as those of true nutritive tissues (Ferreira et al., 2017). Nutritive cells may occur in some Thysanoptera galls (Ananthakrishnan and Raman, 1989), but they are absent in the galls induced by *Nexothrips* studied here. The absence of a true nutritive tissue indicates that the galling *Nexothrips* should feed on epidermal cell contents. Also, the reduced hypersensitive sites next to the oil glands in the galls on *M. splendens* indicate that the *Nexothrips* may access the cells of the oil glands and take advantage of its high energetic content. Taking into consideration the non-occurrence of secretory structures other than the oil glands in leaves of *M. splendens*, we assume their potential for secreting the major portion of the VOCs.

### Potential Roles of the Chemical Profile of the Oil Glands

Currently, the second step of investigation revealed that gall induction and establishment caused alterations in the density and area of the oil glands, which are larger in galls than in the non-galled leaves. The increased size of the oil glands indicates an enhancement in the potential for the production of volatiles in galled condition. Such potential can provide a favorable microenvironment to the galling *Nexothrips* sp., which can benefit from the products of the glands, as proposed for other galling insect-host plant systems (Stone and Schonrögge, 2003).

### Role of Volatiles as Biomarkers and Chemical Signalers

The volatiles can act by chemical signaling for herbivores, and their biosynthesis can be altered in response to herbivory (Valladares et al., 2002; Banchio et al., 2005), as observed for *M. splendens* regarding the concentration of volatiles. Currently, gall induction alters the size and area of the oil glands, and accordingly the concentration of VOCs in the samples of non-galled leaves and of galls is distinct. The substantial changes in the emissions of volatiles as gall induction consequence is expected (Izzo et al., 2006), and is clearly perceived in the content of sesquiterpenes in *M. splendens*. Such quantitative changes have been reported for other three galling herbivore-host plant systems (*cf*. Tooker et al., 2002; Tooker and Hanks, 2004, 2006). The volatiles, besides attracting reproductive partners (Koschier et al., 2000, 2007) may act in direct or indirect plant defenses against natural enemies (Dudareva et al., 2004; Koschier et al., 2007; Oates et al., 2016). Due to the high frequency of galls in the population of *M. splendens* along the year, we can infer that the repellent properties of the sesquiterpenes were not effective against the associated galling *Nexothrips.* The quantitative differences in the content of sesquiterpenes detected by the SPME analysis in *Nexothrips* sp. galls in comparison to the non-galled condition implied favorable features for the galling Thysanoptera. The volatiles produced in the different samples of *M. splendens* should be related both to insect–plant and to plant–plant interactions.

Chemical signaling, mediated by volatiles, may allow the insects to find and recognize their host plants (Hanula et al., 1985; Tooker and Hanks, 2004), but may also attract natural enemies (James and Chem, 2005). Such inference is based on the ability of the inducers to stimulate host plant responses, which trigger local reactions, and may interfere directly with the insect communication with its host plant (Moura et al., 2009a,b; Oates et al., 2016), but also with other plants in the population. In the population of *M. splendens* at the PESV, there is one plant individual, which has never associated to *Nexothrips* sp. The lowest content observed in the chemical profile of this individual of *M. splendens*, the aforementioned control individual, and the highest content observed in the general composite of the non-galled leaves (NGL) of the other individuals in the population indicates that the galling activity of *Nexothrips* sp. may have caused long-distance effects (Mani, 1964) over the population of *M. splendens*. The secondary effects or tele-effects were first described for galls induced in roots, but causing changes in flowers of the host plant, *Heterodera marioni* (Mani, 1964), and has recently been reported for *Ditylenchus* *gallaeformans* galls on *Miconia* spp (Ferreira et al., 2017).

The effect of gall induction on other host plant organs, by the production of secondary metabolites, including volatile compounds, may represent an indirect defense of the plant (Unsicker et al., 2009; Fürstenberg-Hägg et al., 2013; Oates et al., 2016). Despite of their simple molecular structures, the alcohols and aldehydes deriving from the lypoxygenase (LOX) pathway, methyl salicylate, and 3-hexenyl butanoate of low molecular weight pertaining to the GLV class were detected in the samples of *Nexothrips* sp. galls on *M. splendens*, and can act as signaling molecules in plant-herbivore interactions (Yan and Wang, 2006; Damasceno et al., 2010). The chemical signaling between *M. splendens* and its associated galling herbivores may be mediated by some of the terpenes detected in samples of galled leaves. The monoterpenes (geraniol) and the sesquiterpenes may play an attractive role for the adult female of *Nexothrips* sp., as proposed for *Frankliniella occidentalis*, a generalist phytophagous species found worldwide (Koschier et al., 2000, 2007). The geraniol and sesquiterpenes-mediated attraction is yet to be tested for the four galling herbivores reported on *M. splendens* on PESV (Portugal-Santana and Isaias, 2014). The decreasing concentration of δ-muurolene (#67), β-elemene (#58) and α-caryophyllene (#63) in the samples from which the galling thrips were removed (cLRG) could be an indicative that the individuals of *Nexothrips* sp. were manipulating *M. splendens* metabolism and assimilating some of these secondary metabolites.

The detection of methyl salicylate (#42) in the samples of galls had also been related to acquired resistance and indirect plant defense (Oates et al., 2016). Methyl salicylate is a plant semiochemical related to stress signaling (Pickett et al., 2006), and it is generally described as anti-herbivoric, attractive to beneficial insects that would kill herbivores (Bruinsma et al., 2009), and as a pheromone (James and Price, 2004; Troncoso et al., 2012). In *M. splendens*, the production of methyl salicylate neither affected the life cycle of the inducing thrips nor attracted natural enemies, since individuals in several stages of life occurred inside the galls, and the rate of hyperparasitism was apparently low in comparison to other Neotropical systems (Gonçalves et al., 2009; Carneiro et al., 2013). Such inability of methyl salicylate as an anti-herbivore substance may be effect of its low concentration and consequently its limited potential to stimulate the galling thrips responses, crucial for attractiveness or repellency (Koschier et al., 2000, 2002; Bruhin, 2009).

## Conclusion

The rare event of gall induction by the Terebrantia studied here revealed mesophyll compactness and formation of air cavities as new features, first described for the Neotropical Thysanoptera-induced galls. The structural profile of *M. splendens* non-galled leaves and galls revealed that the main alteration regards the number and size of the oil glands. As the only secretory structure differentiated in leaves of *M. splendens*, the oil glands should be the main secretory sites responsible for the peculiar chemical profile of the analyzed samples. The main alteration in GLVs concentration in response to *Nexothrips* sp. activity indicates the GLVs as possible stress biomarkers involved in the host plant-galling Thysanoptera signaling. Moreover, the methyl salicylate in the composite of the non-galled individual reveals a potential plant resistance stimulated as a long-distance effect. In addition to the signaling effects of the volatile compounds produced by the non-galled leaves and the galls on *M. splendens*, it can be hypothesized that the individuals of the galling *Nexothrips* sp. may have captured, incorporated, and metabolized some of these VOCs. This hypothesis is based on the increased levels of some sesquiterpenes detected in the composites containing the galling thrips in comparison to the composites without thrips.

## Author Contributions

NJ and DA did the field sampling. ÉS-S, GS, GLS, and CZ did the chemical analyses. NJ, DA, and RI analyzed the structure. AC did the characterization and ecology of thrips. NJ, ÉS-S, DA, GS, GLS, CZ, AC, and RI analyzed the data and wrote the manuscript.

## Conflict of Interest Statement

The authors declare that the research was conducted in the absence of any commercial or financial relationships that could be construed as a potential conflict of interest.

## Footnotes

1 http://www.microscopia.ufmg.br

2 http://www.metaboanalyst.ca

## Supplementary Material

DATA SHEET S1 Area value of each compound identified in *Myrcia splendens* samples by HS-SPME-GC/MS.

## Abbreviations

cLRG: clean-leaf rolling galls, where cLRG stands for clean leaf galls
Ctrl: control individual
LRGwT: leaf rolling galls without thrips, where “wT” stands for “with thrips”
NGL: non-galled leaves
